# The Employer's Liability

**Published:** 1921-05-21

**Authors:** 


### MEDICAL AID FOR DOMESTIC SERVANTS.

### The Employer's Liability.

According to the common law a master is under no legal obligation to provide his servant with medicine or medical attendance during illness. This applies to all servants. There is no exception to the rule even in the case of illness due to injury received in the master's service, or in the case of servants who reside in the master's house. The only exception is in regard to apprentices residing with their master—a condition of things practically unknown to-day—and young domestic servants.

An instance of this was the case of Jeffrey *v.* Donald (1901), an action of damages for negligence at common law, raised by the mother of a domestic servant against her employer. The servant in question was a young girl of thirteen years of age. She had fallen down a stair, and was so badly injured as to become unconscious. The defendant did not send for a doctor, and two days later the girl died. The ground of decision was important in respect of the girl's youth and her being under the *quasi* guardianship of the defendant, for it is a well-known principle of the law of master and servant that a master has a duty of protection towards young domestic servants resident in family with him.

One of the features of the common law, however, as contrasted with statute law is that it reflects current public opinion; gradually and insensibly the common law advances so as to come in line with current thought and feeling on political, economic, and social questions.

We notice in a recent case in the Scotch Courts, McKeating *v.* Frame (1921), that the duty of an employer as to procuring medical aid for his servant has been rather emphasised, and the decision, in our opinion, effects an important qualification on the common law principle we have stated. This was also an action for damages against the employer by the father of a girl of seventeen years of age, who had been employed by the defendant as a domestic servant in the neighbourhood of the town of Shotts, Lanarkshire. The case as set out for the plaintiff was that she was a strong and healthy girl, and but for a cold she did not complain of illness. Next day she felt severe pain in the chest. She then informed both the defendant and his wife thereof, but they took no steps to ascertain her condition or to have her treated medically. She received no further attention from them, but continued with her work, though steadily getting worse, for three days. She then collapsed in the byre, where she was found in a very weak condition. She was carried into the farmhouse unconscious. The defendant next morning went to her bedroom, saw her condition, and ordered her to get up and go home, and said to her that there was no one to look after her. Though she endeavoured then to obey the order of the defendant, her master, she was unable to do so owing to bodily weakness. In the afternoon of the same day, however, in order to carry out the defendant's instructions to go home, she, by a supreme effort, managed to get up and dress and walk down to the road-end, where she got the 4 p.m. 'bus to a point from which she walked to her mother's house. At the time she left the farm the girl was almost in a dying condition, and traversed with extreme difficulty the distance of half a mile between the farm and the place where she could meet the omnibus. Before she left the farm, and throughout the journey, her face was pale, her lips cyanosed, her movements extremely feeble. On the following day, March 26, 1919, she died of double pneumonia and influenza. On the day before her death the girl, in the presence of a justice of the peace, emitted and signed a dying declaration narrating the circumstances of her removal from the defendant's house.

The judge of first instance dismissed the action on the ground that there was no case to go to a jury, but on appeal the Inner House of the Court of Session have recalled this judgment and allowed an issue for jury trial. Of course, it is for the jury to determine the facts, and the plaintiff's narrative of them may be unduly coloured; but meantime the result of the decision is that the plaintiff has stated a relevant case in law.

The Lord Justice-Clerk thus dealt with the duty of the defendant in regard to procuring medical aid: "Nor can I agree with the Lord Ordinary's views as to the master's duty to provide medical attendance for a servant who is unwell. It was, perhaps, quite true that a master was not bound in law to provide medical attendance for his sick servant, but nowadays all he requires to do is to notify the panel doctor. It is averred that on the Monday, the day on which the defendant ordered the girl to leave for home, he was in Shotts himself. I do not know whether the panel doctor resided in Shotts; I should think that is very likely. At any rate, he could easily have sent word to the panel doctor that there was at his farm a sick person entitled to get medical assistance."

So far as we know, that is the farthest that any judge has gone in regard to the duty we are discussing, and we think it not inappropriate to take notice of the case, although the decision given deals only with a preliminary legal point.

